# Brachytherapy-Integrated Hyperthermia for Pelvic Malignancies: Clinical Evidence, Technical Platforms, Thermal Dosimetry, and Translational Barriers

**DOI:** 10.3390/cancers18182984

**Published:** 2026-09-15

**Authors:** Yuanjie Cao, Ronglan Cui, Chen Li, Youheng Tan, Qingxin Wang, Wei Wang, Jaeho Cho, Jie Chen

**Affiliations:** 1 Department of Radiation Oncology, Tianjin’s Clinical Research Center for Cancer, Key Laboratory of Cancer Prevention and Therapy, Tianjin Medical University Cancer Institute & Hospital, National Clinical Research Center for Cancer, Tianjin 300060, China; cyjro325@tjmuch.com (Y.C.); lichen.radonco@tmu.edu.cn (C.L.); qwang01@tmu.edu.cn (Q.W.); wangwei1625@tjmuch.com (W.W.); 2 Department of Radiation Oncology, Yonsei University College of Medicine, Seoul 03722, Republic of Korea; choiyr322@yuhs.ac; 3 School of Automation, Central South University, Changsha 410083, China; tyheng@csu.edu.cn

**Keywords:** brachytherapy, hyperthermia, thermobrachytherapy, pelvic malignancy, thermal dosimetry, thermometry, treatment planning, quality assurance, evidence map

## Abstract

Brachytherapy-integrated hyperthermia combines implant-based radiation delivery with localized heating, but the evidence spans heterogeneous diseases, devices, temperature measurements, and study designs. We conducted a systematic search-based evidence map with revision-stage record reconciliation, human adjudication, report-to-study linkage, and design-matched clinical appraisal. Of 8189 unique records screened in the original search, 2178 candidate or uncertain records underwent documented revision-stage human adjudication. During acceptance-stage revision, all 3919 records originally assigned to the machine exclusion stratum also received a primary human title/abstract re-screen decision; 3916 exclusions were confirmed and three boundary records were reclassified. The final unified worklist comprised 285 records. Ninety-two verified/supporting report units were adjudicated (15 direct clinical, 34 technical/platform, 24 supporting, and 19 excluded). The 15 core clinical reports represented 14 independent studies and generated 17 appraisal records using RoB 2, ROBINS-I, and JBI tools. Two randomised phase III trials did not show improved disease control or survival with added hyperthermia; both were judged to raise some concerns for risk of bias, and both had important thermal intervention limitations. One non-randomised cervical comparison was at serious risk of bias from confounding by indication. Uncontrolled prostate, anal, gynaecologic, and mixed pelvic series supported procedural feasibility but not causal efficacy. Two predefined supplemental saturation batches added no new eligible evidence in any family or domain. The evidence supports standardised multipoint thermometry, measurement-anchored spatial reconstruction, coupled planning, platform qualification, and disease-specific trials only after thermal fidelity gates are passed.

## Simple Summary

Hyperthermia may increase radiation sensitivity, while brachytherapy provides a direct route for implant-compatible heating and temperature sensing. We systematically mapped the clinical and technical evidence for this combined strategy in pelvic malignancies and reconstructed the selection pathway during major revision. The clinical core comprised 15 reports representing 14 independent studies. Two randomised trials did not demonstrate clinical benefit from the historical regimens as delivered; most of the remaining evidence was non-comparative. Technical feasibility has advanced faster than clinical validation, and limited spatial thermometry and inconsistent thermal dose reporting remain the principal barriers. The most defensible near-term strategy is therefore not immediate routine adoption, but staged development: a minimum reporting dataset, platform qualification, thermally verified workflow studies, and only then disease-specific comparative trials.

## 1. Introduction

Brachytherapy delivers steep, conformal dose gradients through intracavitary or interstitial applicators. The same implant geometry can also provide access for local heating, temperature sensors, cooling, or directional energy delivery. This physical co-localization is the defining attraction of brachytherapy-integrated hyperthermia; radiation and heat can be coordinated within one anatomically constrained treatment workflow rather than delivered as unrelated modalities [1,2].

The biological rationale is temperature-, duration-, dose-, and sequence-dependent. Subablative heating can increase perfusion and oxygenation, inhibit repair of radiation-induced DNA damage, intensify protein and membrane stress, and directly injure cells in hypoxic, acidic, nutrient-deprived, or otherwise radio-resistant tumour compartments. Immune and vascular effects may contribute but are context-dependent. Radiosensitization is generally strongest when heat and radiation are simultaneous or closely sequenced, although a short interval cannot compensate for inadequate spatial coverage or insufficient thermal dose [3,4,5,6,7,8,9].

In this review, oncologic hyperthermia denotes controlled subablative heating, commonly around 39–45 °C, intended to radiosensitize and stress tumour tissue without planned coagulative destruction. Thermal ablation intentionally produces irreversible tissue destruction at substantially higher temperature–time exposures. Because biological injury depends on both temperature and exposure time, no single temperature is an absolute boundary. Ablation-oriented studies were used only as explicitly labelled transferable engineering evidence when they informed implant geometry, sensing, cooling, material compatibility, or coupled planning [7].

Previous literature has demonstrated numerous applicators and heating concepts, but technical feasibility and clinical efficacy are not equivalent. This evidence map therefore asks four linked questions: what direct clinical evidence exists; which platform families have been technically validated; how temperature and thermal dose were measured or reconstructed; and what implementation gates must be passed before disease-specific trials are justified?

## 2. Materials and Methods

### 2.1. Review Design and Reporting Framework

We conducted a systematic search-based, staged evidence map rather than a conventional meta-analysis. Evidence mapping was selected because the literature includes clinical studies, engineering prototypes, phantom and computational studies, preclinical models, QA reports, protocols, and conference abstracts that cannot be combined into a single effect estimate. Reporting was aligned with PRISMA 2020 where applicable and with evidence map methodology [10,11]. The review was not prospectively registered. During major revision, we reconstructed the protocol and amendment history from dated project files; all revision-stage rules are labelled as retrospective methodological enhancements rather than original prespecifications. The reconstructed protocol and dated amendment/deviation log are provided in the Supplementary Protocol.

### 2.2. Information Sources and Searches

PubMed, Embase, Scopus, and Web of Science Core Collection were searched without initial year, language, or document-type limits. Search paths combined brachytherapy terms with induced hyperthermia, historical thermoradiotherapy terminology, pelvic disease terms, implant-compatible devices, thermometry, thermal dose, planning, and QA. Publication-facing search tables provide each path as a complete standalone expression with field tags, platform, date, and raw count; numbered search history shorthand is retained only in the audit crosswalk.

### 2.3. Original Screening and Revision-Stage Reconciliation

The searches identified 17,463 records. After within-database deduplication, 15,493 records entered cross-database deduplication; 7304 duplicates were removed, and 8189 unique records were screened. The original workflow used machine-assisted title/abstract classification to assign 932 records to a broad candidate stratum, 1246 to a maybe/unclear reserve, 2037 to background/citation chasing, 3919 to machine exclusion, and 55 to a duplicate/nonrepresentative record stratum. These were prioritization or record management strata, not final human eligibility decisions. During acceptance-stage revision, all 3919 records in the machine exclusion stratum underwent human title/abstract re-screening, with one primary human decision recorded per record across four reviewers. Potentially relevant records were escalated for report-level assessment and full-text verification where available. Human review confirmed exclusion for 3916 records and identified three boundary records: one BT-compatible technical report and two historical/supporting thermoradiotherapy reports, which were reclassified and incorporated into the revised evidence framework. An earlier archived 100-record AI-assisted sample is retained only as developmental calibration evidence because complete principal investigator sign-off was not preserved.

During revision, the 2178 candidate and uncertain reserve records were partitioned among four reviewers (CYJ 500, CRL 500, LC 500, WQX 678), with one primary human decision per record. Disagreements and 48 QC overrides were resolved by documented senior review and consensus; the process was not described as four independent screens of every record. Human review initially advanced 331 records. Quality control retained 306, and removal of 27 exact or nonrepresentative copies yielded 279 unique candidates. Two direct additions and one corrective addition produced the earlier 282-record unified worklist. The complete acceptance-stage human re-screening of the separate 3919-record machine exclusion stratum then rescued three additional records, yielding the final 285-record worklist.

### 2.4. Evidence Gap Definition, Targeted Retrieval, and Stopping Rule

An evidence gap was operationally defined as an unpopulated or inadequately characterized cell in a domain-by-context matrix comprising disease site, study design, heat–brachytherapy integration class, platform family, thermometry coverage, thermal dose reporting, coupled planning/validation, and QA/implementation. A report could close a gap only when it contributed extractable information to that cell. This rule was established during revision to replace the original subjective phrase “sufficiently characterized.”

The targeted branch contained 4082 source-gated records, 924 gap term hits, and a bounded 58-record technical/supporting worklist. All 58 received an adjudicated disposition. The earlier 19-report/20-record set was a drafting and retrieval priority subset, not the endpoint of the supplemental process. The three must-retrieve records were subsequently resolved: two verified full reports were added as Include-B technical evidence, while one distinct historical conference report identity remained unretrieved and was closed because later full reports covered the platform route. Eight additional reports remained optional recommended retrievals, 32 were covered by more informative platform family reports, and 15 were background closures. Retrieval did not stop merely because a preferred drafting set had been reached. The operational stopping rule required completion of predefined database, backward/forward citation, device/team, and study family searches plus two consecutive logged batches yielding no new eligible clinical study, report family, platform family, thermometry method, thermal dose method, planning method, or QA domain. This revision-stage rule was defined and frozen before S1 and S2 were executed. S1 screened 1012 deduplicated platform/device/thermometry records and identified one additional potentially eligible but family-covered bibliographic report identity; S2 screened 733 deduplicated clinical/historical/family closure records and identified two additional potentially eligible but family-covered identities. None was added as a new verified synthesis unit because each was already represented by a more informative report from an existing family/domain. Neither batch added a new evidence family or domain, so the stopping criterion was met on 8 September 2026. Unretrieved reports were recorded as such and were never converted into full-text exclusions.

### 2.5. Eligibility and Evidence Hierarchy

Include-A comprised direct clinical evidence in pelvic malignancies in which heating was integrated with the brachytherapy implant, applicator, catheter, source channel, or tightly coordinated treatment workflow. Include-B comprised direct BT-compatible platform, thermometry, thermal dose, planning, preclinical, or QA evidence, including non-pelvic platforms with explicit transferability. Include-C comprised supporting historical, mechanistic, protocol, registry, or abstract-level evidence that informed interpretation but did not contribute independent clinical efficacy. Exclusions included EBRT-plus-hyperthermia without a defined BT-integrated role, ablation without a transferable engineering contribution, non-oncologic or non-pelvic applications without transferability, reviews without a necessary citation role, and duplicates without unique information.

The synthesis hierarchy was: randomised/comparative clinical evidence; non-comparative clinical feasibility and safety; technical/platform evidence; thermometry, thermal dose, and planning evidence; phantom/computational/preclinical evidence; abstracts; and linked reports. Technical feasibility was never used as evidence of clinical efficacy. Conference abstracts were tabulated separately and were not given definitive full-text risk-of-bias judgments.

### 2.6. Data Extraction, Report Linkage, and Appraisal

We extracted disease and setting, study design, analysis denominator, comparator, brachytherapy regimen, heating modality and device, timing, temperature target, thermometry geometry, thermal dose metric, follow-up, efficacy, toxicity, planning, QA, and evidence locator. Reports were linked to study families using author group, site, recruitment period, sample size, intervention details, registry/protocol linkage, and cohort overlap. The 15 core clinical reports represented 14 independent studies; the early prostate umbrella report was retained as supporting evidence and not counted again.

Clinical appraisal was design-matched: RoB 2 for randomised trials, ROBINS-I (2016) for the non-randomised comparative intervention study, the JBI Case Series checklist for uncontrolled series, and the JBI Case Report checklist for a single case [12,13,14,15]. JBI judgments used Yes/No/Unclear/NA without numerical scores. Thermal intervention fidelity and applicability were assessed separately from internal validity. Thus, inadequate heat delivery did not automatically produce a high risk-of-bias judgment, and adequate heating did not remove confounding. Technical evidence was appraised descriptively across validation setting, BT component and compatibility, device/heating characterisation, thermometry or model validation, reported thermal metrics, repeatability and uncertainty, safety and limitations, and clinical transferability. These domains were recorded in Supplementary Table S4; no unvalidated composite engineering score was calculated.

### 2.7. Synthesis

Statistical pooling was not attempted because diseases, comparison structures, brachytherapy regimens, heat delivery platforms, thermal measurements, and endpoint definitions were heterogeneous. Results were summarized by design and disease, then cross-linked to platform, thermometry, thermal dose, planning, and QA evidence. Readiness labels are qualitative descriptors of evidence maturity and are not GRADE ratings.

During revision, generative AI-assisted tools (ChatGPT [GPT-5.6 Sol] and Codex [GPT-5.6 Sol]; OpenAI, San Francisco, CA, USA) were used to assist with record organization, consistency checking, and drafting support. These tools did not independently determine study eligibility, generate primary research data, or make final methodological judgments. All publication-facing screening decisions, evidence classifications, extracted results, risk-of-bias judgments, figures, and manuscript content were reviewed and verified by the authors.

## 3. Results

### 3.1. Study Selection and Evidence Accountability

**Figure 1:**
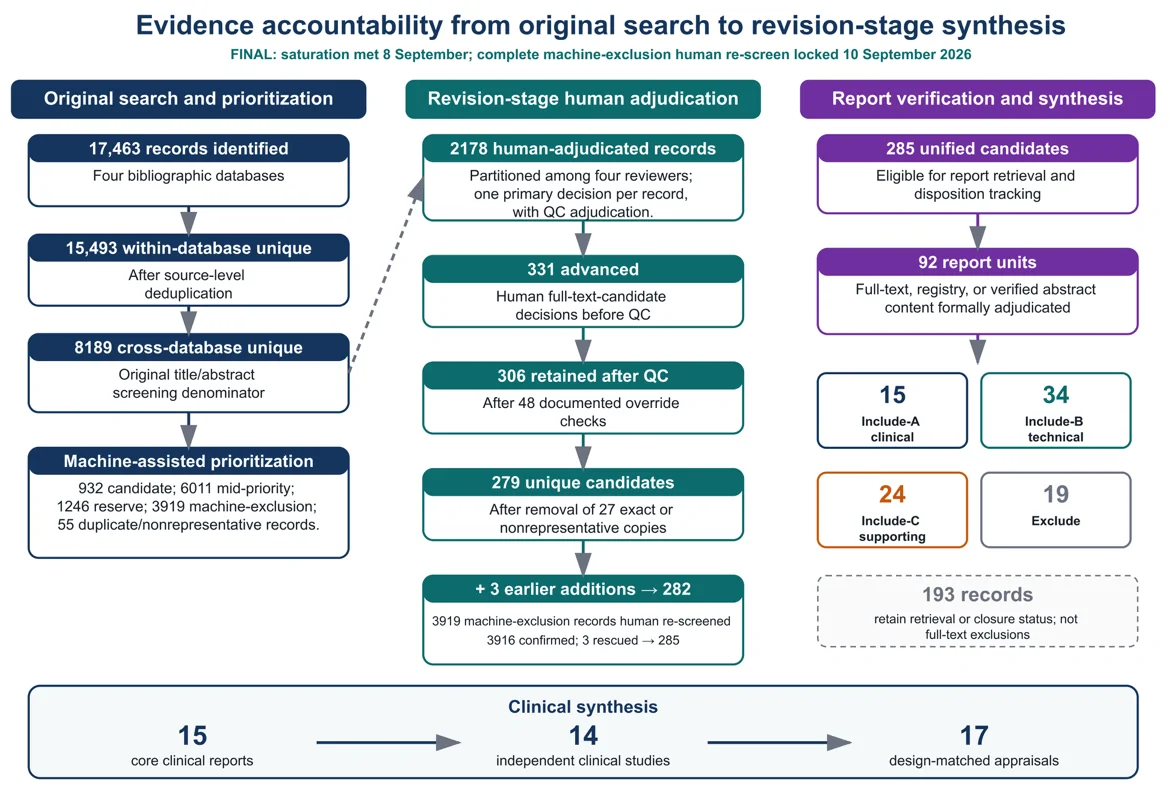
Revision-stage evidence accountability flow. The diagram separates original machine-assisted prioritization from revision-stage human decisions and distinguishes verified/supporting report adjudication from records retaining retrieval or closure status. Counts are locked as of 10 September 2026. All 3919 original machine exclusion records received a primary human title/abstract re-screen decision; 3916 exclusions were confirmed, and three records were reclassified. Two consecutive predefined supplemental batches produced zero new family/domain yield, and the stopping criterion was met.

Figure 1 shows the corrected distinction between screening strata, human adjudication, verified/supporting report units, and documented retrieval/disposition decisions. Of the 285 unified candidates, 92 report units had full-text, registry, or sufficiently verified abstract content available for formal adjudication: 15 were direct clinical (Include-A), 34 technical/platform (Include-B), 24 supporting (Include-C), and 19 excluded. The remaining 193 records were not treated as full-text exclusions; each retained a retrieval or closure status. Complete human re-screening of the 3919-record machine exclusion stratum confirmed 3916 exclusions and rescued one technical and two historical/supporting reports. Separately, the two predefined revision-stage supplemental saturation batches screened 1012 and 733 deduplicated records. Three additional bibliographic identities encountered in those batches were already represented by more informative reports from existing families/domains and were not added as new synthesis units. Neither saturation batch added a new eligible clinical study, report family, BT-compatible platform family, thermometry method, thermal dose method, planning method, or QA domain. The predefined revision-stage stopping criterion was therefore met.

### 3.2. Clinical Evidence: 15 Reports Representing 14 Studies

The clinical evidence cited in this section comprised 15 core reports plus two linked or supporting reports [16,17,18,19,20,21,22,23,24,25,26,27,28,29,30,31,32]. The 15 core reports mapped to two randomised phase III trials, one non-randomised comparative cervical study, ten uncontrolled case series, and one case report; the early mixed prostate umbrella report did not add an independent study [22]. Seventeen appraisal records were completed: four RoB 2 outcome-level judgments, two ROBINS-I outcome-level judgments, ten JBI case series appraisals, and one JBI case report appraisal. The prospective protocol and historical mixed-site supporting report were retained as linked or contextual reports rather than counted as independent studies [20,25]. The characteristics of the 14 independent core clinical studies are summarized in Table 1.

### 3.3. Randomised and Comparative Evidence

The cervical phase III trial randomised 224 patients and analysed 205. Three-year disease-free survival was approximately 0.67 with brachytherapy alone and 0.60 with brachytherapy plus interstitial hyperthermia; corresponding local control was approximately 0.84 and 0.88, without a significant advantage. Efficacy and serious/late-toxicity results were judged as having some concerns, principally because the analysis was not strict intention-to-treat and no accessible prespecified analysis plan was identified. Thermal characterisation relied on three thermocouples and emphasized maximum temperature rather than spatial coverage or cumulative thermal dose. The correct clinical interpretation is therefore challenging but balanced: the trial provides evidence against benefit from the historical regimen as delivered, while not proving that a modern, adequately planned and spatially verified intervention is ineffective.

The RTOG phase III trial accrued 184 patients and analysed 173 evaluable patients with mixed recurrent or persistent tumours. It did not show a significant improvement in response, local control, or survival. Both outcome sets raised concerns. Only one patient met the study’s minimum heating adequacy criteria, a major treatment fidelity failure that limits mechanistic interpretation but is analytically distinct from risk of bias. The earlier non-randomised cervical comparison was at serious risk of bias because physicians preferentially assigned hyperthermia to patients with larger tumours or poorer response to external beam radiotherapy.

### 3.4. Disease-Specific Synthesis and Readiness

Cervical/gynaecologic disease has the strongest direct randomised evidence, but benefit has not been established. Prostate cancer has the most mature contemporary uncontrolled series, including prospective multicentre salvage experience, but no randomised comparison. Anal cancer remains historical proof-of-concept, and other pelvic sites are supported mainly by very small or mixed-site reports. BT-adjacent or mixed-modality clinical reports were retained only as supporting context when direct implant-level integration was absent or incompletely separable [33,34]. Readiness therefore differs by disease: no site currently has both reproducible thermal fidelity evidence and definitive comparative efficacy. These disease-specific evidence-readiness assessments and corresponding next steps are summarized in Table 2.

### 3.5. Technical Platforms and Clinical Cross-Linking

**Figure 2:**
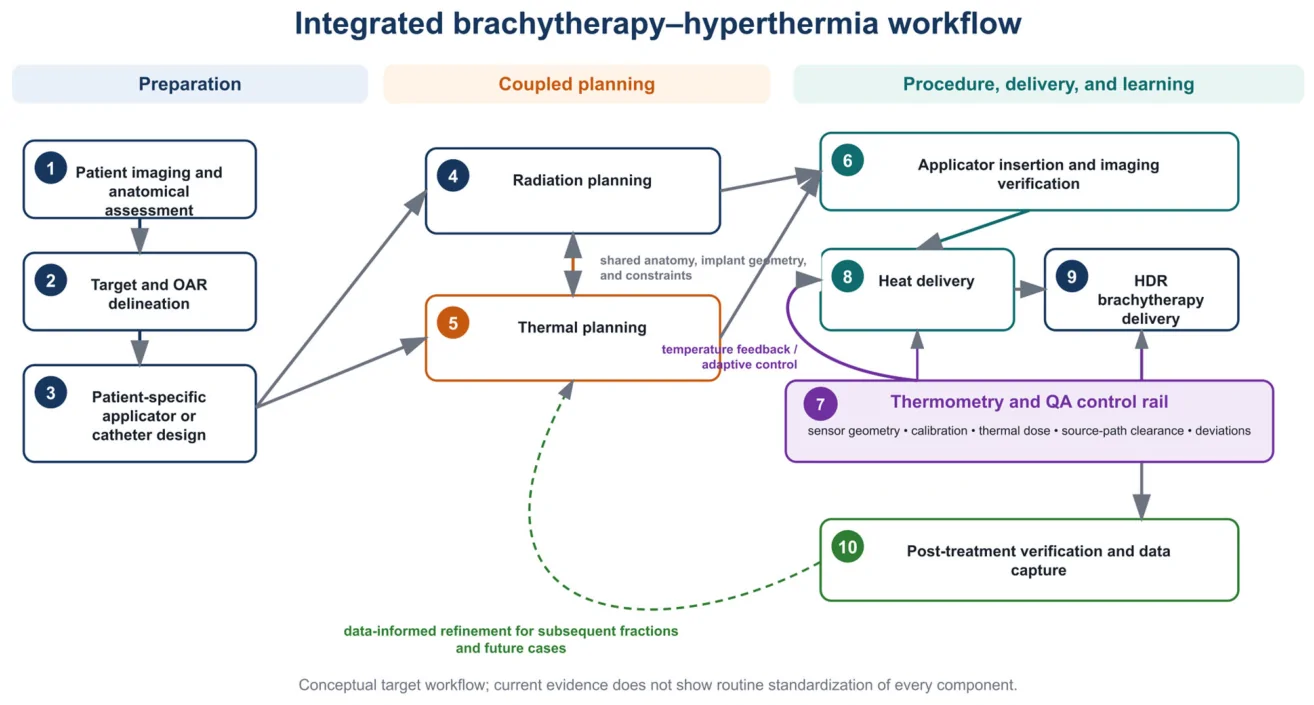
Conceptual workflow for brachytherapy-integrated hyperthermia. Shared anatomy and implant geometry should drive coupled radiation and thermal planning, with thermometry and QA spanning device verification, heat delivery, source delivery, and post-treatment data capture.

The platform literature can be grouped into radiofrequency, microwave, ultrasound, warm water/intraluminal, and magnetic or ferromagnetic implant concepts. Integrated catheter concepts appeared early: Patel and colleagues used bioheat modelling to optimize dual- and triple-heater conductive interstitial catheters and proposed placing radioactive sources, including iridium seeds, within gaps between heater elements and delivering them through the hollow catheter structure, creating a shared architecture for simultaneous interstitial thermoradiotherapy [35]. This anticipated contemporary multifunctional platforms but remained an engineering/modelling study rather than a clinically validated system. Table 3 cross-links each family to the clinical sites, measurement strategy, principal limitation, and immediate development need; the full platform-level crosswalk remains supplementary. These principal technical configurations and their integration with brachytherapy are illustrated in Figure 2.

### 3.6. Thermometry and Thermal Dosimetry

Thermometry remained the central methodological bottleneck. Historical studies often reported one or a few point measurements, maximum temperature, or device set-point rather than spatial coverage. CEM43 and temperature volume metrics provide a more interpretable temperature–time framework, but they remain sensitive to sensor position and cannot recover unmeasured cold regions [6,40,45,55,68]. The principal thermometry strategies, their advantages and limitations, and their best-fit clinical contexts are summarized in Table 4.

**Table 4:** Comparative framework for pelvic thermometry strategies.

Strategy	Advantages	Limitations	Best-Fit Context	Current Recommendation
Multipoint invasive probes	High temporal resolution; immediately compatible with many implants	Sparse spatial sampling; probe displacement; electromagnetic perturbation	Cervix/prostate/anal implants with planned sensor tracks	Most feasible near-term standard; report geometry and uncertainty
MR thermometry	Potential volumetric coverage without additional invasive tracks	Susceptibility and RF artifacts, motion, reference drift, device compatibility, cost and access	Selected MRI-compatible pelvic platforms	Promising, not universal; require motion/artifact validation
Model-assisted reconstruction	Full-field estimate; supports temperature volume analysis, planning, and adaptive control. Patient validation achieved approximately 1.3 °C mean absolute sensor-level error [68].	Dependent on tissue properties, perfusion, geometry registration, boundary conditions, calibration, and sampling. Mean temperature may be reproduced better than spatial heterogeneity.	All sites when imaging and device geometry are known	Use as a measurement-anchored reconstruction; validate both point agreement and cold region/temperature volume coverage before clinical decisions.
Hybrid measurement model	Combines measured anchors with spatial reconstruction	Higher workflow and computational burden; uncertainty propagation required	Development programmes and multicentre qualification	Preferred long-term strategy after platform-specific validation

For currently available interstitial cervical and prostate workflows, the most realistic short-term strategy is multipoint invasive thermometry with prespecified geometry, sensor calibration, normal-tissue monitoring, and thermal dose reporting. Patient-specific reconstruction should augment rather than replace measurement until validated. In a prostate MECS-IHT validation study, model-assisted reconstruction expanded sparse invasive measurements into three-dimensional temperature volume information and achieved a mean absolute measured–simulated difference of 1.3 ± 1.1 °C across 641 applicator–sensor comparisons; however, the model reproduced mean temperature better than spatial heterogeneity and depended on geometry registration and simplified perfusion assumptions [68]. MR thermometry is most attractive where the applicator, implant materials, and workflow are demonstrably MRI-compatible and pelvic motion can be corrected.

### 3.7. Planning, Quality Assurance, and Reporting

Most reports optimized radiation and heating separately. A clinically credible platform should instead use common target/OAR contours, implant coordinates, device constraints, predicted thermal coverage, sensor trajectories, and feedback thresholds [40,56,57,58,67,69,70]. Interstitial hyperthermia QA should include applicator characterisation, power output verification, temperature–sensor calibration, electromagnetic compatibility, channel/source clearance, thermal safety limits, and prospective recording of interruptions and failed delivery [41,42]. A phantom study using a 13.56 MHz RF electrode inside a 5-Fr microSelectron HDR catheter showed that an air-filled catheter–electrode gap produced unstable SAR distributions and hotspots, whereas water filling stabilized resonance and yielded reproducible SAR profiles across repeated trials, electrode lengths, and insertion depths [43]. This provides a concrete, measurable platform qualification test, although clinical sterility, leakage, and in vivo coverage still require validation. Contemporary disease-specific image-guided brachytherapy standards should define the radiation backbone so that any incremental thermal effect is interpretable [1,2,71,72].

## 4. Discussion

### 4.1. Principal Interpretation

This revised evidence map shows a clear asymmetry: the field has demonstrated multiple ways to generate heat within or around a brachytherapy implant, but it has not established a reproducible clinical benefit. The two randomised trials are the highest-level and most challenging evidence. Their negative results should neither be dismissed as mere delivery failures nor overextended into proof that an adequately dosed modern intervention cannot work. They show that the historical regimens as delivered did not improve outcomes, while their limited thermal characterisation prevents a strong mechanistic conclusion.

Uncontrolled studies add feasibility, toxicity, and workflow information but do not repair the efficacy gap. The prostate literature is the most mature among recent series, whereas cervical cancer has the most direct randomised evidence. Neither is ready for routine adoption because one lacks randomised comparative validation and the other lacks evidence that a spatially adequate, reproducible thermal intervention improves contemporary image-guided brachytherapy outcomes.

Early reports also described combined interstitial hyperthermia and brachyradiotherapy and heterogeneous Phase I/II thermoradiotherapy experience [73,74]. These reports improve historical completeness but do not enlarge the core clinical denominator: incomplete disease-specific reporting, mixed treatment techniques, uncertain cohort relationships, and limited source detail precluded quantitative extraction or additional risk-of-bias appraisal.

### 4.2. How a Future Definitive Trial Should Differ

A new phase III trial should not begin with a novel device alone. It should follow technical qualification and a prospective workflow study demonstrating reproducible thermal coverage. The definitive trial should use one disease and setting, a common contemporary image-guided brachytherapy backbone, preimplant thermal-delivery eligibility, prespecified sensor coverage and thermal dose/coverage targets, platform and centre credentialing, central real-time or near-real-time QA, intention-to-treat primary analysis, and prespecified supportive fidelity and thermal dose–response analyses. Failure to reach the thermal target must be retained as a protocol delivery outcome, not used to remove randomised participants. Endpoints should include disease-specific local control, patient-reported morbidity, grade ≥3 late toxicity, and procedural burden.

### 4.3. Prioritized Translational Roadmap

**Figure 3:**
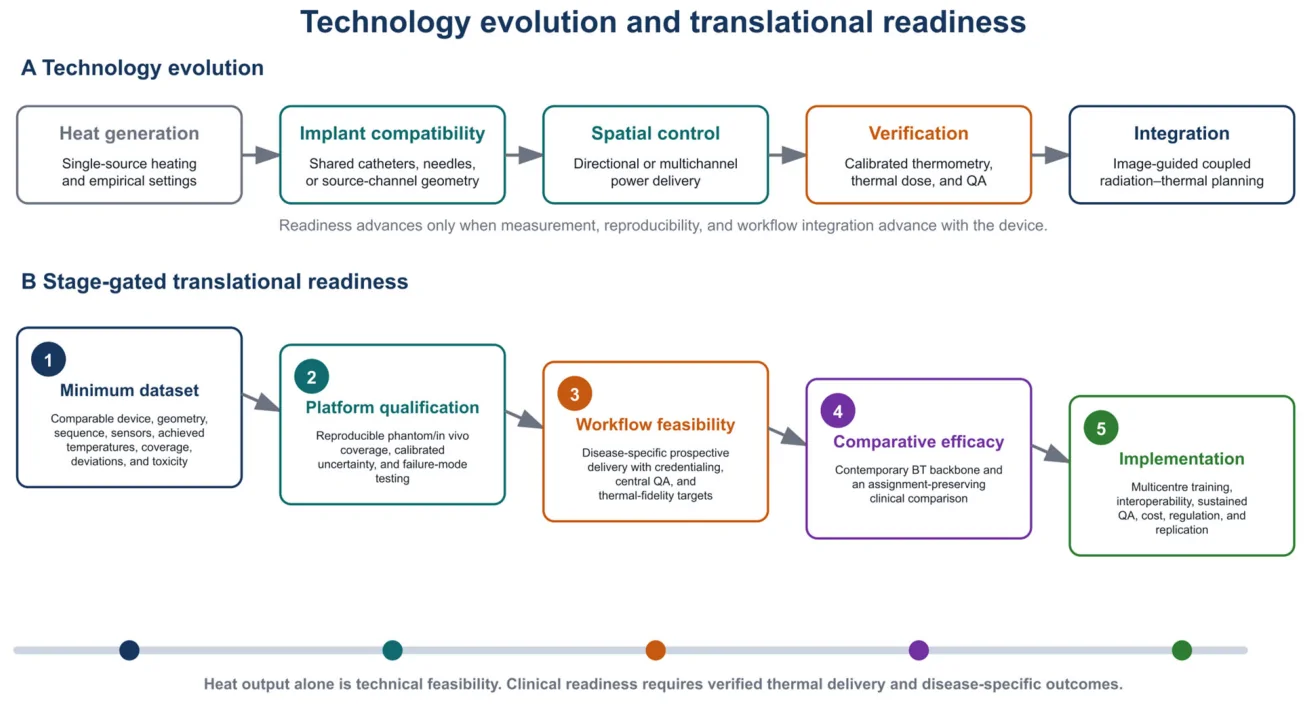
Evolution and translational readiness of brachytherapy-integrated hyperthermia. Readiness reflects verified measurement, integrated workflow, prospective clinical validation, and implementation capacity rather than heat generation capability alone.

The translational pathway should therefore proceed through sequential, stage-gated development rather than device-driven escalation. As summarized in Table 5 and Figure 3, priorities progress from standardized reporting and platform qualification to prospective workflow validation, comparative efficacy testing, and ultimately multicentre implementation. Advancement between stages should depend on predefined evidence of thermal fidelity, reproducibility, safety, and clinical feasibility rather than heat-generation capability alone.

**Table 5:** Stage-gated development roadmap. Progression requires passing the preceding gate; device novelty alone is insufficient.

Priority Stage	Time Horizon	Deliverable	Measurable Gate	Decision Enabled
1. Minimum dataset	0–12 months	Consensus fields for device, geometry, sequence, sensors, achieved temperatures, CEM43/coverage, interruptions, and toxicity	≥90% completeness in a prospective registry	Makes studies comparable
2. Platform qualification	6–24 months	Bench/phantom and in vivo validation, calibration, uncertainty and failure mode testing	Reproducible coverage and safety within predefined tolerance	Allows multicentre technical transfer
3. Workflow feasibility	12–36 months	Prospective disease-specific study with coupled planning, credentialing and central QA	Prespecified thermal fidelity and procedural feasibility targets met	Justifies comparative testing
4. Comparative efficacy	After Stage 3	Randomised or otherwise rigorous disease-specific comparison on a contemporary BT backbone	Clinically meaningful local-control/toxicity target met on ITT analysis	Tests incremental clinical value
5. Implementation	After efficacy	Multicentre training, interoperability, cost and regulatory programme	Sustained QA performance and external replication	Supports routine use

### 4.4. Limitations

This review was not prospectively registered, and the original selection pathway used machine-assisted prioritization that was insufficiently documented for reproducible eligibility decisions. We therefore reconstructed the protocol, completed human adjudication of the candidate and uncertain strata, documented retrieval/closure decisions, and separated verified/supporting reports from unretrieved records. During acceptance-stage revision, all 3919 records originally assigned to machine exclusion were also human-re-screened; three boundary records were reclassified. No record in that stratum therefore remained dependent solely on the machine-generated decision. These steps improve accountability but do not erase the retrospective nature of the reconstruction. Residual human screening or classification error remains possible, as in any evidence synthesis. Historical terminology, non-English records, abstract-only evidence, inaccessible reports, and overlapping study families remain potential sources of error. The evidence map is systematic and audited but should not be described as exhaustive. Technical evidence was assessed with a transparent domain framework rather than a validated universal engineering risk-of-bias instrument. The predefined saturation criterion was met after two consecutive zero-family/domain-yield batches; this stopping decision does not establish that the evidence base is exhaustive.

## 5. Conclusions

Brachytherapy-integrated hyperthermia is biologically plausible and technically feasible, but clinical effectiveness is unproven. Two randomised trials did not demonstrate a significant clinical benefit from the historical regimens as delivered, and the remaining clinical evidence is predominantly small and non-comparative. The field’s immediate priority is reproducible thermal measurement and workflow qualification—not another efficacy claim based on device output or sparse maximum temperatures. Multipoint invasive thermometry with standardised geometry is currently the most implementable option; validated hybrid measurement–model approaches may ultimately provide the spatial coverage needed for adaptive control. Disease-specific comparative trials should proceed only after platforms demonstrate reproducible thermal coverage, integrated planning, and auditable QA.

## Figures and Tables

**Table 1:** Concise characteristics of the 14 independent clinical studies. Full brachytherapy regimens, follow-up, outcomes, locators, and item-level appraisal are reported in Supplementary Tables S5 and S6.

Report	Disease/Site	*N*	Design	BT–HT Integration	Thermometry/Dose	Efficacy	Toxicity	Appraisal
Kapp et al., 1994 [18] (GLOBAL-00047)	Anal cancer	14	Phase I/II single-arm prospective clinical study	Warm water IHT immediately after HDR using the same needles; goal 42.5 °C for 40 min	Three-point thermocouple in 1–2 extra needles; manual mapping every 0.5–1 cm	cCR 11/14; pathologic CR 10/14	Combined treatment generally well tolerated; complications reported and discussed	JBI Case Series
Seegenschmiedt et al., 1993 [24] (GLOBAL-00057)	Localized implantable pelvic tumours	26	Clinical case series	915 MHz microwave IHT immediately before Ir-192 placement and after source removal, 45–60 min at 41–44 °C	Extensive invasive 3D thermometry; T90/T50/T25/Tmin-derived metrics and quality-of-heating analyses	3-mo CR 17/26; 12-mo LC 16/21; 8/26 locoregional relapse/regrowth	Acute effects 8/26; long-term complications 7/26; thermal damage linked to high Tmax	JBI Case Series
Shimm et al., 1990 [27] (GLOBAL-00070)	Mixed tumours; substantial pelvic subset	72 total; 49 pelvic	Historical single-arm clinical series	RF 0.5 MHz or microwave 915 MHz IHT; most heated intraoperatively for 30 min, aiming Tmin 42 °C	Multiple intratumoral temperature measurements; Tmin/Tmax analysed	CR 25/69 evaluable; CR associated with tumor volume, dose, and Tmin	17 complications; 9 severe, all in pelvic tumours	JBI Case Series
Kukielka et al., 2014 [19] (GLOBAL-00099)	Locally recurrent prostate cancer	25	Retrospective single-arm salvage cohort	IHT immediately before each BT fraction; 41–43 °C for 60 min; 62/75 planned sessions delivered	Thermistors through interstitial catheters; prostate/periurethral temperatures monitored	2-y biochemical control estimate 74%; all alive at report; 7 biochemical failures	No grade ≥3 toxicity; grade 2 rectal bleeding in 1; late toxicity in 22 with >6 mo follow-up	JBI Case Series
Zolciak-Siwinska et al., 2013 [16] (GLOBAL-00106)	Locally advanced cervical cancer	224 randomised; 205 analysed	Randomised Phase III trial	500 kHz RF; same tandem/needles as BT; 4 sessions; 45 min before and during HDR-BT; ≥30 min >42.5 °C intended	Three thermocouples in tandem/needles; only maximum temperatures recorded; reported 42–49 °C depending tolerance	3-y DFS 0.67 BT vs. 0.60 BT + HT; *p* ≈ 0.178. 3-y LC 0.84 vs. 0.88; *p* ≈ 0.991	No significant acute/late toxicity difference; cumulative 3-y grade 3+ late toxicity ~0.168 vs. 0.166, *p* = 0.692	RoB 2
Zolciak-Siwinska et al., 2012 [23] (GLOBAL-00148)	Locally advanced cervical cancer	76 analysed	Prospective non-randomised comparative cohort/Phase I	500 kHz RF; same BT applicators; 4 sessions; 45 min before/during BT; >42.5 °C, up to ~49 °C; ≥30 min heating before BT begins	Three thermocouples; maximum temperatures only; no correction for self-heating, with spot checks when RF is off	5-y DFS 73.6% BT vs. 65.8% BT + HT; 5-y LC 89% vs. 83%	No significant early/late toxicity difference; late rectal/bladder/vaginal events reported by RTOG/EORTC and SOMA	ROBINS-I (2016)
Kukielka et al., 2016 [21] (GLOBAL-00150)	Primary prostate cancer	76	Retrospective single-arm cohort	IHT before every HDR fraction; target 41–43 °C for 60 min	≥8 antennas and ≥4 thermometers recommended; intraprostatic temperatures reported	Clinical/biochemical outcomes reported; study focused on tolerance	No grade 3–4 acute GU/GI; late grade 2 GU ≤ 23.7%; no late grade ≥2 GI	JBI Case Series
Emami et al., 1996 [17] (GLOBAL-00156)	Recurrent/persistent mixed tumours; ~42–43% pelvic	184 accrued; 173 evaluable	Randomised Phase III trial	1–2 HT sessions; target minimum measured tumour temp 43 °C for 60 min; HT before or before + after implant; interval RT-HT ≤ 60 min	Multiple intratumoral thermometry tracks recommended; protocol adequacy criteria later defined; only 1 patient met minimum adequacy criteria	No significant difference in response/local control/survival; abstract CR ~53% vs. 55%; 2-y survival ~34% vs. 35%	Acute grade 3–4 toxicity 12% vs. 22%; late grade 3–4 toxicity 15% vs. 20%; differences not significant	RoB 2
Strnad et al., 2026 [30] (GLOBAL-00185)	Locally recurrent prostate cancer	109	Prospective multicentre single-arm Phase II	IHT within 1 h before or during every BT fraction/series; target ≥ 40–41 °C for 60 min	Interstitial temperature monitoring; target coverage described; detailed thermal parameters in methods	5-y bDFS 58.9%; metastasis-free 77.7%; CSS/OS reported	Serious late toxicity low; no grade ≥2 late rectal toxicity; urinary grade 3 events uncommon	JBI Case Series
Rafla et al., 1989 [26] (GLOBAL-00186)	Mixed tumours with pelvic subset	38 patients; 46 lesions; 18 pelvic lesions	Historical clinical case series	Interstitial HT delivered around BT; repeated heating sessions and modal thermal dose concept	Thermistors in heating antennae/catheters; ~960 temperature points per case analysed	Overall complete regression 54% in 35 evaluable lesions; site-specific data presented	Treatment complications reported; fibrosis/poor healing discussed	JBI Case Series
Sorbe et al., 1990 [31] (GLOBAL-00192)	Vaginal squamous-cell carcinoma	9	Clinical case series	Two HT sessions after first/last intracavitary treatment; 60 min ≥42.3 °C; 30–60 min RT–HT interval	Thermistors at tumour periphery/applicator; SAR phantom work; directional temperature curves	Nine treated, but detailed response reported only for 2 illustrative cases	Two detailed cases reported without major treatment complications; full 9-patient toxicity not provided	JBI Case Series
Parisi et al., 1990 [32] (GLOBAL-00215)	Vaginal recurrence of cervical carcinoma	3	Clinical case series	Intracavitary microwave HT in conjunction with Ir-192; 3–4 HT sessions around 41.5–43 °C	Intracavitary applicator/temperature treatment values reported	2 CR, 1 PR	Mild cystitis/proctitis/vaginal bleeding/bleeding cystitis reported by case	JBI Case Series
Boggs et al., 2015 [29] (GLOBAL-00224)	Pelvic recurrence of bladder cancer	1	Case report	Interstitial microwave hyperthermia delivered with implanted catheters during HDR treatment course	Thermal sensors in treatment catheters; CT-based spatial temperature mapping; hot spots documented	No evidence of growth at 8 mo; clinical follow-up at 10 mo	Treatment tolerated well; no major reported acute treatment complication	JBI Case Report
Piotrkowicz et al., 2005 [28] (GLOBAL-05221)	Recurrent endometrial/cervical cancer	10	Clinical case series	1–2 interstitial HT procedures using same rigid metal needles; BT started without interrupting HT once target temp reached; 45–60 min, up to 46–49 °C	Temperature target and concurrent delivery described; limited formal thermal dose metrics	Complete vaginal tumour regression 8/10	Grade 1 rectal/bladder reactions in 6; no late side effects reported; one later sigmoid perforation judged outside reirradiated volume	JBI Case Series

Abbreviations: BT, brachytherapy; HT, hyperthermia; IHT, interstitial hyperthermia; JBI, Joanna Briggs Institute; RoB, risk of bias. An uncontrolled study can support feasibility and safety characterisation but not causal efficacy.

**Table 2:** Disease-specific evidence strength and next translational requirement.

Disease Group	Evidence Base	Efficacy Interpretation	Thermal Limitation	Readiness/Next Step
Cervical/gynaecologic	One randomised Phase III cervical trial, one earlier non-randomised comparative Phase I study, and several small vaginal/gynaecologic series.	The randomised cervical study did not demonstrate a clear DFS or local-control advantage from adding interstitial hyperthermia to HDR brachytherapy. The earlier non-randomised study is seriously confounded by indication.	Thermometry was sparse in the randomised/Phase I cervical program (three thermocouples, maximum-temperature emphasis); CEM43/spatial thermal coverage were not robustly characterized.	Clinical readiness remains investigational; future trials require disease-specific prospective validation with standardised thermal dose/coverage reporting.
Prostate	One primary-treatment retrospective series and two distinct salvage cohorts, including a later prospective multicentre Phase II study.	The strongest uncontrolled evidence is in salvage prostate treatment, where long-term biochemical control and acceptable toxicity have been reported.	Interstitial thermometry and repeated IHT integration are better described than in many gynaecologic studies, but thermal dose standardization remains incomplete.	Potentially the most clinically mature disease-specific application among the uncontrolled series, but comparative validation is still required.
Anal cancer	One small Phase I/II single-arm study (14 patients).	High complete-response rates were reported with combined EBRT, HDR implant, and interstitial warm water hyperthermia, but the series is small and noncomparative.	Evidence remains feasibility-level rather than definitive efficacy evidence.	Useful historical direct pelvic proof-of-concept; insufficient for routine adoption.
Other pelvic/mixed-site	Includes two mixed-site historical series with pelvic subsets, one RTOG randomised trial, one pelvic implant series and a bladder case report.	The RTOG randomised trial did not show clear benefit, but only one patient met minimum protocol thermal adequacy criteria, indicating profound intervention-fidelity failure.	Older studies contain valuable thermometry/thermal dosimetry information but are heterogeneous in devices, dose, timing, and reporting.	Best interpreted as historical/technical support rather than disease-specific efficacy evidence.

**Table 3:** Platform family comparison and disease-specific clinical relevance.

Platform Family	Clinical/Site Linkage	Strength	Principal Limitation	Required Next Step
Interstitial RF and conductive catheters [35,36,37,38,39,40,41,42,43]	Cervix, vagina, prostate, mixed pelvis	Direct heating through implant needles/electrodes; historically closest to clinical integration. A 5-Fr microSelectron catheter study demonstrated a measurable interface-QA solution for repetitive RF heating [43].	Spatial non-uniformity, electrode coupling, sparse sampling, and catheter--electrode air gaps can destabilize resonance and create hotspots.	Geometry-aware power control; prespecified sensor coverage; source-channel clearance; and platform-specific repeatability testing of the catheter–electrode interface.
Interstitial microwave [24,26,27,44,45,46]	Prostate, vaginal/pelvic and mixed-site series	Catheter-compatible antennas; repeated heating feasible	Hot spots, antenna coupling, catheter/after loader compatibility	Central QA for antenna output, cooling, and source-channel clearance
Warm water/intraluminal [18,47]	Anal and intracavitary concepts	Simple, low-cost, physically compatible with hollow needles/applicators	Limited depth and directional control; temperature may not represent tumour volume	Use only for shallow, geometrically accessible targets with mapped coverage
Catheter ultrasound [48,49,50,51,52,53,54,55,56,57,58]	Primarily prostate/cervix engineering and pilot evidence	Directional sectors, cooling, and patient-specific control	Complex hardware, acoustic shadowing, registration and workflow burden	Prospective closed-loop validation against independent thermometry
Magnetic/ferromagnetic seeds or nanoparticles [59,60,61,62,63,64,65,66,67]	Mainly computational, phantom, and preclinical	Potentially source-like implant geometry and self-regulation	Field access, material characterisation, eddy currents, retrieval/regulatory issues	Standardised material, field, dose, safety, and explantability testing

## Data Availability

This review generated new record-level screening decisions, retrieval/disposition data, report-to-study linkage, clinical extraction, and design-matched appraisal datasets. The publication-facing versions are supplied as Supplementary Tables S1–S7. Audit-level working files containing provenance and adjudication fields are available from the corresponding author on reasonable request.

## Supplementary Materials

The following supporting information can be downloaded at: https://www.mdpi.com/article/10.3390/cancers18182984/s1, Table S1: standalone database search strategies, search history crosswalk, saturation logs, and acceptance-stage machine exclusion human re-screen audit; Table S2: record-level screening, retrieval and disposition ledger for 285 unified candidates; Table S3: report-to-study family linkage, identity resolution, and abstract-only evidence; Table S4: 34-report technical-platform extraction and reliability domains; Table S5: 17 clinical appraisal records with signaling-question/item-level rationale; Table S6: complete 14-study clinical extraction; Table S7: clinical–technical crosswalk and final numerical truth table; Supplementary Protocol, retrospectively reconstructed protocol and dated amendment/deviation log.

## Abbreviations

The following abbreviations are used in this manuscript:

BT	Brachytherapy
CT	Computed Tomography
CEM43	Cumulative Equivalent Minutes at 43 °C
EBRT	External Beam Radiotherapy
HDR	High-Dose-Rate
MRI	Magnetic Resonance Imaging
OAR	Organ at Risk
PRF	Proton Resonance Frequency
QA	Quality Assurance
RF	Radiofrequency
T50	Temperature achieved in 50% of the target volume
T90	Temperature achieved in 90% of the target volume
