# Supplementary material for: Brachytherapy-Integrated Hyperthermia for Pelvic Malignancies: Clinical Evidence, Technical Platforms, Thermal Dosimetry, and Translational Barriers
Source: Cancers (Basel). 2026 Sep 15;18(18):2984. doi: 10.3390/cancers18182984 (PMC13604313; doi:10.3390/cancers18182984)
Supplement: Supplementary file 1 [file cancers-18-02984-s001.zip › Supplementary Protocol.pdf]

Supplementary Protocol and Amendment Log

Retrospectively reconstructed during major revision; not prospectively registered

This document consolidates the revision-stage screening/selection protocol and clinical appraisal protocol. It records what was original, what was reconstructed from dated files, and what was added during revision. It must not be described as a prospectively registered protocol.

Generative-AI-assisted tools supported record organization, consistency checking, and drafting assistance during revision. They did not independently determine eligibility or make final evidence or risk-of-bias judgments. All publication-facing decisions and text were reviewed and verified by the authors.

Revision-Stage Screening and Selection Protocol

Status and purpose

This protocol was created during major revision on 2026-09-02 and amended through 2026-09-10 to correct and complete the staged evidence-selection pathway. It was not prospectively registered and must not be described as an original prospectively specified protocol. It operationalizes the existing review scope, distinguishes retrieval priority from eligibility, and records revision- and acceptance-stage methodological amendments.

Review question

What clinical, technical-platform, thermometry, thermal-dosimetry, treatment-planning, and quality-assurance evidence informs brachytherapy-integrated or explicitly brachytherapy-adjacent hyperthermia for pelvic malignancies, and what barriers limit translation into a reproducible modern clinical workflow?

Evidence roles

1. Direct clinical BT+HT evidence — patients with pelvic malignancy receive brachytherapy and hyperthermia in the same treatment pathway, with sufficient information to characterize both components.

2. Direct BT-compatible technical evidence — heating device, applicator, seed, catheter, source channel, thermometry system, or planning method explicitly designed for or tested with brachytherapy compatibility.

3. Direct thermal/planning evidence — thermometry, thermal dose, or thermal planning is explicitly linked to a brachytherapy-compatible catheter, applicator, implant, or combined workflow.

4. QA/implementation evidence — evidence directly addressing sensor behavior, source-transfer interaction, combined-device safety, calibration, workflow, or platform-specific QA.

5. Transferable background evidence — non-brachytherapy, non-pelvic, ablation, foundational thermal-dose, or general hyperthermia evidence that supports a specific mechanism, limitation, measurement principle, or engineering transferability question. These records may be cited but are not counted as independent BT-integrated clinical-efficacy or platform evidence.

6. Conference abstract/minimal evidence — abstract-only evidence retained separately for mapping or citation linkage.

7. Linked/overlapping evidence — companion reports, overlapping cohorts, duplicate platform reports, or linked method papers retained without independent counting.

Title/abstract decisions

- Include — full text required: direct or potentially direct evidence; uncertainty cannot be resolved from title/abstract.
- Background/citation chasing: relevant foundational or transferable evidence but not independent BT-integrated evidence.
- Conference abstract/minimal: abstract-only signal requiring separate tracking.
- Duplicate/linked: probable duplicate or companion report requiring linkage.
- Exclude: fails the scope and has no defined background role.
- Unclear — full text required: insufficient information with plausible direct relevance; handled conservatively as requiring full text.

Inclusion codes

- I1: pelvic clinical treatment pathway includes both brachytherapy and hyperthermia.
- I2: device/platform explicitly integrates or is compatible with BT sources, channels, applicators, catheters, needles, seeds, or afterloading.
- I3: thermal dosimetry, thermometry, or planning explicitly linked to BT-compatible or combined interstitial/intracavitary geometry.
- I4: combined-workflow QA, safety, calibration, or implementation evidence.
- I5: companion/linked report with a unique method, outcome, or follow-up contribution.
- I6: insufficient abstract information but a credible direct-integration signal.

Background codes

- B1: foundational thermal dose or temperature–time evidence.
- B2: thermobiology or radiation–heat sequencing evidence.
- B3: transferable catheter/interstitial heating or thermometry technology without explicit BT integration.
- B4: MR thermometry, PRF-shift artifact, motion, material-compatibility, or reconstruction evidence relevant to the proposed workflow.
- B5: disease-specific RT+HT evidence in which BT is present but the heating intervention is not integrated with or linked to BT delivery.
- B6: non-pelvic thermobrachytherapy platform with a specific transferable component.
- B7: guideline, reporting, or QA background.

Exclusion codes

- E1: no hyperthermia, thermotherapy, thermal monitoring, or relevant thermal method.
- E2: no brachytherapy and no explicit transferable platform, thermometry, planning, QA, or foundational role.
- E3: EBRT plus hyperthermia only, with no BT component and no specific background role.
- E4: thermal ablation only, without a defined transferable BT/thermometry/planning role.

- E5: non-pelvic application without explicit transferability.
- E6: benign/non-oncologic application without explicit transferability.
- E7: review, editorial, commentary, or general background without a needed citation role.
- E8: duplicate with no unique contribution.
- E9: abstract or record contains insufficient extractable information and no reliable linkage.
- E10: unrelated intervention, anatomy, disease, outcome, or technology.

Hyperthermia–ablation boundary

Mild/moderate oncologic hyperthermia is intended to enhance radiotherapy through temperature- and time-dependent biological interactions without planned coagulative destruction. Thermal ablation is intended to produce direct tissue destruction at substantially higher thermal exposure. Ablation studies are not clinical efficacy evidence for thermobrachytherapy. They may be retained only as transferable background when they directly inform catheter geometry, implant compatibility, MR thermometry, cooling, material artifact, thermal planning, or another explicitly named combined-workflow issue.

Second-stage review population

1. All 932 records originally categorized as Include for full-text review.

2. All 1,246 records originally categorized as Maybe/Unclear.

3. All 58 records in the final targeted technical/supporting worklist. The earlier 19-report/20-record set was a drafting and retrieval-priority subset rather than the stopping endpoint.

4. The 55 records in the original duplicate/nonrepresentative-record stratum were resolved through bibliographic identity and deduplication tracking rather than clinical eligibility screening. This stratum is distinct from the 55 non-must-retrieve records within the later 58-record technical/supporting worklist.

5. Stratified audit samples from the original Exclude and Background/Citation chasing strata.

Workflow

1. Apply the above codes to title and abstract without using final manuscript inclusion as the decision rule.

2. Preserve the current-publication status only as an audit field; it must not determine revision-stage eligibility.

3. Records with plausible direct evidence or unresolved direct relevance proceed to full text.

4. Records retained only as background are not counted in the independent evidence denominator.

5. All assisted decisions require author verification before being described as final human decisions.

6. Disagreements or uncertain records are escalated to a senior clinical/physics author and resolved by documented consensus.

Validation and completion standard

- Human title/abstract adjudication is required for all 932 candidate-pool records and all 1,246 Maybe/Unclear reserve records. Assisted decisions organize the queue but do not close a record.

- Verify all decisions that would remove a currently included publication record and all missing-abstract, historical-terminology, ablation-boundary, duplicate/linked, and conference-only records.
- All potentially direct or unresolved records proceed to documented full-text retrieval attempts and full-text eligibility adjudication.
- Archived stratified samples were created during development of the original machine-exclusion and background/citation-chasing strata. The 100-record AI-assisted preliminary sample included 40 proposed-exclusion records, but complete principal-investigator sign-off was not preserved; it is therefore retained only as developmental/calibration evidence and is not the principal machine-exclusion QC evidence.
- Acceptance-stage amendment dated 10 September 2026: all 3,919 records originally assigned to the machine-exclusion stratum underwent human title/abstract re-screening. Records were distributed across CYJ (980), CRL (980), LC (980), and WQX (979), with one primary human decision per record; this was not independent duplicate screening of all records. Potentially relevant records were escalated to report-level assessment and full-text verification where available. Human review confirmed exclusion for 3,916 records and reclassified three boundary records: GLOBAL-01685 as Include-B technical/platform evidence and GLOBAL-02406 and GLOBAL-04221 as Include-C historical/supporting evidence. This acceptance-stage procedure was not part of the original protocol and must not be represented as prospectively planned.
- Report the size, reviewer, date, and outcome of every verification population.
- Do not describe the assisted process as independent human dual screening.

Supplemental stopping rule

The 58-record targeted branch will be completed rather than stopped at the original 19-record Priority A set. Future gap-directed retrieval may stop only when:

1. every targeted candidate has a recorded decision and reason;

2. all direct or unresolved records have undergone full-text retrieval attempts;

3. two consecutive review batches add no new eligible clinical study, report family, BT-compatible platform family, thermometry method, thermal-dose method, planning method, or QA domain;

4. unresolved evidence gaps remain explicitly labelled as gaps rather than being treated as filled; and

5. the decision and date are recorded in the amendment log.

Consolidated amendment/deviation log

Date	Amendment	Reason	Effect
2026-09-02	Reclassified the 932-record step as a candidate stratum rather than completed full-text review	The original path reflected prioritization, not final eligibility	Required complete revision-stage adjudication and a corrected flow diagram
2026-09-02	Added complete review of the 1,246 maybe/unclear reserve	High-sensitivity reserve had not been fully adjudicated	Reduced false-exclusion risk

Date	Amendment	Reason	Effect
2026-09-02	Required disposition of all 58 targeted candidates	The earlier 19-record subset was drafting oriented	Created must-retrieve, recommended, family-covered, and background-closure queues
2026-09-02	Added design-matched clinical appraisal	Peer review requested evidence-reliability assessment	Introduced RoB 2, ROBINS-I and JBI tools while separating thermal fidelity
2026-09-03	Locked reviewer partition and QC workflow	Reviewer provenance needed explicit documentation	Recorded CYJ/CRL/LC/WQX batches and consensus overrides
2026-09-07	Locked report-to-study linkage and clinical appraisal	Duplicate/overlap resolution was completed	15 core reports became 14 independent studies and 17 appraisal records
2026-09-08	Froze and executed saturation Batch S1	Challenge remaining platform/device/thermometry gaps using database, citation and device/team routes	1,012 deduplicated records; 1 additional eligible but family-covered technical report identity; 0 new family/domain
2026-09-08	Executed saturation Batch S2 and met the stopping rule	Challenge clinical/historical terminology and close known study/platform families independently of S1	733 deduplicated records; 2 additional eligible but family-covered report identities; second consecutive 0 new family/domain
2026-09-10	Completed human title/abstract re-screen of all 3,919 machine-exclusion records	Acceptance-stage reviewer requested direct clarification and strengthening of machine-exclusion quality control	3,916 exclusions confirmed; GLOBAL-01685 reclassified Include-B and GLOBAL-02406/GLOBAL-04221 Include-C; final worklist 285 and report units 92; 14 studies and 17 appraisals unchanged

Revision-stage clinical risk-of-bias protocol

Date: 2026-09-02

Purpose

This protocol governs the revision-stage appraisal of the clinical evidence in the review. It was added in response to peer review and is reported as a revision-stage methodological enhancement; it was not part of the original search protocol.

The appraisal deliberately separates three questions that must not be collapsed into one judgment:

1. Internal validity: could the estimated clinical effect be biased by study design, conduct, analysis, or reporting?

2. Thermal-intervention fidelity: was hyperthermia delivered, measured, and reported well enough to establish that a biologically meaningful thermal intervention was actually tested?

3. Applicability to brachytherapy-integrated hyperthermia: was heat physically and temporally integrated with the brachytherapy implant/workflow, merely present elsewhere in the radiotherapy pathway, or transferable only as background evidence?

Inadequate thermometry or low achieved temperature can limit intervention fidelity and interpretation without automatically making a randomized comparison high risk of bias. Conversely, apparently adequate heating does not repair confounding in a non-randomized study.

Unit of appraisal

The unit is the independent clinical study, not the publication. Linked reports, protocols, registries, conference abstracts, and expanded/overlapping cohorts are mapped to their parent study and are not counted as independent outcome evidence unless non-overlap is demonstrated.

Design-specific appraisal tools

The tool choice follows the actual analytic design. A publication labelled “prospective,” “phase II,” or “pilot” is not treated as comparative unless it contains an eligible comparison that can estimate the effect of adding hyperthermia.

RoB 2 domains

Randomized trials are assessed for:

1. bias arising from the randomization process;

2. bias due to deviations from intended interventions (effect of assignment to intervention is the primary estimand);

3. bias due to missing outcome data;

4. bias in measurement of the outcome; and

5. bias in selection of the reported result.

The algorithm-informed overall judgment is retained. Departures from the algorithm, if any, require a written rationale. Primary judgments focus on the principal tumour-control or survival outcome at the clinically most relevant reported time point; toxicity is assessed separately when its risk-of-bias profile differs materially.

ROBINS-I domains

Non-randomized comparative studies are assessed for:

1. confounding;

2. selection of participants;

3. classification of interventions;

4. deviations from intended interventions;

5. missing data;

6. measurement of outcomes; and

7. selection of the reported result.

The target trial is the same disease-specific treatment comparison with random allocation to otherwise identical brachytherapy pathways with or without the prespecified hyperthermia intervention. Prespecified key confounders include tumour size/volume, stage and risk group, prior irradiation, indication (primary versus salvage), implant geometry, radiation dose and fractionation, concurrent systemic treatment, calendar period, performance status, and factors used by clinicians to select hyperthermia.

JBI case-series and case-report judgments

For case series, item-level judgments cover explicit inclusion criteria, reliable and consistently applied condition measurement, consecutive and complete inclusion, demographic and clinical reporting, outcome/follow-up reporting, and appropriate statistical analysis. For case reports, appraisal covers patient description, history/timeline, diagnostic assessment, intervention, post-intervention condition, adverse events, and lessons supported by the case.

These checklists determine whether a descriptive report is sufficiently credible to characterize feasibility or safety. They do not convert an uncontrolled series into evidence of comparative efficacy.

Separate thermal-intervention fidelity assessment

Each clinical study receives the following non-RoB descriptors:

- Integration class: I, implant/workflow-integrated; II, brachytherapy-adjacent multimodality; III, transferable background only.
- Heat–radiation timing: simultaneous, specified sequential interval, same day but interval unclear, different day, or unclear.
- Thermometry coverage: multipoint/spatial, limited point sampling, surrogate/model only, or absent/unclear.
- Thermal-dose reporting: CEM43 or equivalent dose-volume metric; temperature summary only; target set point/device output only; or absent.
- Protocol delivery: proportion receiving assigned heat, treatment duration, target temperature, achieved temperature/dose, interruptions, and reasons for non-delivery, when reported.
- Fidelity judgment: adequate for the intended mechanistic claim; partially characterized; inadequate/failed delivery; or indeterminate.

Thermal fidelity is interpreted as an intervention-exposure and applicability issue. Any efficacy analysis restricted to patients who achieved a thermal threshold is labelled supportive/per-protocol and is not substituted for the intention-to-treat randomized estimate.

Overall synthesis rules

1. Comparative-effect conclusions are led by randomized evidence, with design-specific risk-of-bias judgments stated alongside estimates.

2. Non-randomized comparisons are not pooled with randomized trials and are interpreted primarily for consistency, feasibility, and hypothesis generation.

3. Uncontrolled cohorts and case reports support feasibility, delivery, toxicity signals, and workflow characterization, but not causal efficacy claims.

4. Abstract-only and limited-access reports are tabulated separately and do not receive definitive full-text appraisal.

5. Overlapping cohorts are clustered; the most informative report is the primary source and linked publications do not increase the independent-study denominator.

6. Disagreements or unverifiable items are recorded as Unclear/No information, never inferred as favourable.

Reproducibility and verification

The revision working file records, for every judgment, the study identifier, design, appraisal tool, domain/item response, supporting quotation or page/table location, reviewer rationale, and overall judgment. AI-assisted preliminary judgments were not treated as final until author verification; all publication-facing locked judgments were subsequently reviewed and verified by the authors. The manuscript will report the tool, design mapping, independent-study unit, and the separation of bias from thermal fidelity and applicability.

Locked revision-stage denominators

Item	Locked value
Original unique records screened	8,189
Human-adjudicated candidate/reserve records	2,178
Unified full-text candidate worklist	285
Verified report units adjudicated	92
Include-A / Include-B / Include-C / Exclude	15 / 34 / 24 / 19
Core clinical reports	15
Independent clinical studies	14
Clinical appraisal records	17

Final technical saturation disposition

The final technical retrieval triad was resolved on 8 September 2026. GLOBAL-01470 and GLOBAL-02436 were verified and added as Include-B technical reports; GLOBAL-06705 remained a distinct historical conference-report identity without a verified full report and was closed because later full reports covered the platform route. These changes updated the report denominator from 87 to 89 and Include-B from 31

to 33. The acceptance-stage complete human re-screen on 10 September 2026 then added GLOBAL-01685 as one verified Include-B technical report and GLOBAL-02406 and GLOBAL-04221 as two Include-C historical/supporting reports. GLOBAL-04221 was reconciled as the same bibliographic identity as alternate background-pool record GLOBAL-05398 and was counted once. The final unified worklist therefore contains 285 records: 92 verified/supporting report units (15 Include-A, 34 Include-B, 24 Include-C, 19 Exclude) and 193 retrieval/closure records that are not full-text exclusions. The clinical denominator remains 15 core reports representing 14 independent studies and 17 appraisal records. Separately, the two predefined saturation batches screened 1,012 and 733 deduplicated records and added no new eligible family/domain; the stopping criterion was met on 8 September 2026. The later human-rescreen amendment strengthened screening accountability but did not retrospectively alter the predefined saturation rule.

Final saturation-batch summary

Batch	Search routes	Records screened	New eligible report identities	New family/domain	Yield
S1	Platform/device/thermometry database search + citation/device-team chasing	1,012	1 (family-covered)	0	Zero
S2	Clinical/historical terminology + family/citation chasing	733	2 (family-covered)	0	Zero

Operational interpretation: "zero" refers to zero newly represented eligible evidence family/domain. Newly encountered duplicates, reports already covered by an existing family, transferable background, and otherwise non-map-changing records remain visible in the record-level audit ledger. The complete S1/S2 record ledgers and adjudication rules are retained with the revision audit files.
